# Implantation of Embryonic Cardiomyocytes in a Post-Infarction Myocardium: A Long-Term Outcome after 25 Years of Follow-Up

**DOI:** 10.26502/acmcr.96550583

**Published:** 2023-03-09

**Authors:** Yu L Schevchenko, VG Gudymovich

**Affiliations:** 1 St. George Clinic of Thoracic and Cardiovascular Surgery, Pirogov National Medical and Surgical Center; 70 Nizhnyaya Pervomayskaya St., Moscow, 105203, Russia

**Keywords:** Coronary Heart Disease, Cell Replacement Therapy, Implantation of Embryonic Cardiomyocytes

## Abstract

In this article, we report the first case of embryonic cardiomyocyte implantation in a patient with severe ischemic heart lesions. In 1998, a 58-year-old male underwent coronary artery bypass grafting supplemented by injection of a pre-prepared suspension of human embryonic cardiomyocytes. During the next 24 years, the patient felt well and reported quite satisfactory quality of life. He is currently 82 years old and also feels well. He has no signs of circulatory insufficiency. No complications associated with cell implantation were observed during the entire follow-up period.

## 1. Background

Organ and tissue transplantation has become a milestone in medicine of the 20^th^ century. Many technological and medical studies were aimed to develop such a method. The development of the autojector idea proposed by S.S. Bryukhonenko resulted in the creation of heart-lung devices, or artificial blood circulation. Transplantation of kidneys, heart, liver and other organs has already become a routine clinical procedure. Cell cultures have become a new step in this direction. It seemed as if the main challenge for partial organ replacement was about to be addressed, when implantation of cell cultures would become an easy alternative to organ transplantation. Allogeneic transplantation of pancreatic islet culture [1], extracorporeal use of donor isolated hepatocytes [2], application of keratinocyte or fibroblast cultures, and a number of other studies have raised hopes in the research society. Moreover, experiments with pluripotent embryonic cells were very promising in terms of growing new organs in the near future [3]. However, new obstacles have emerged, such as compatibility between the donor and the recipient, suppression of the immune response against the transplanted tissue to avoid its rejection, ethical issue. Most importantly, it is yet impossible to create ideal conditions sufficient to cultivate cellular structures outside an organism.

## 2. Methods

### 2.1 Preparation of the Case

Our team got involved in this interesting, laborious, and mysterious work and achieved intriguing and unexpected results within a short period of time [4,5]. We performed experiments on:

- cultivation of embryonic cardiomyocytes and their implantation into the damaged myocardium;
- endotheliocyte cultures used to prevent thrombosis in vascular grafts and calcification of prosthetic valves;
- Stimulation of angioneogenesis in ischemic tissues.

**Figure 1:**
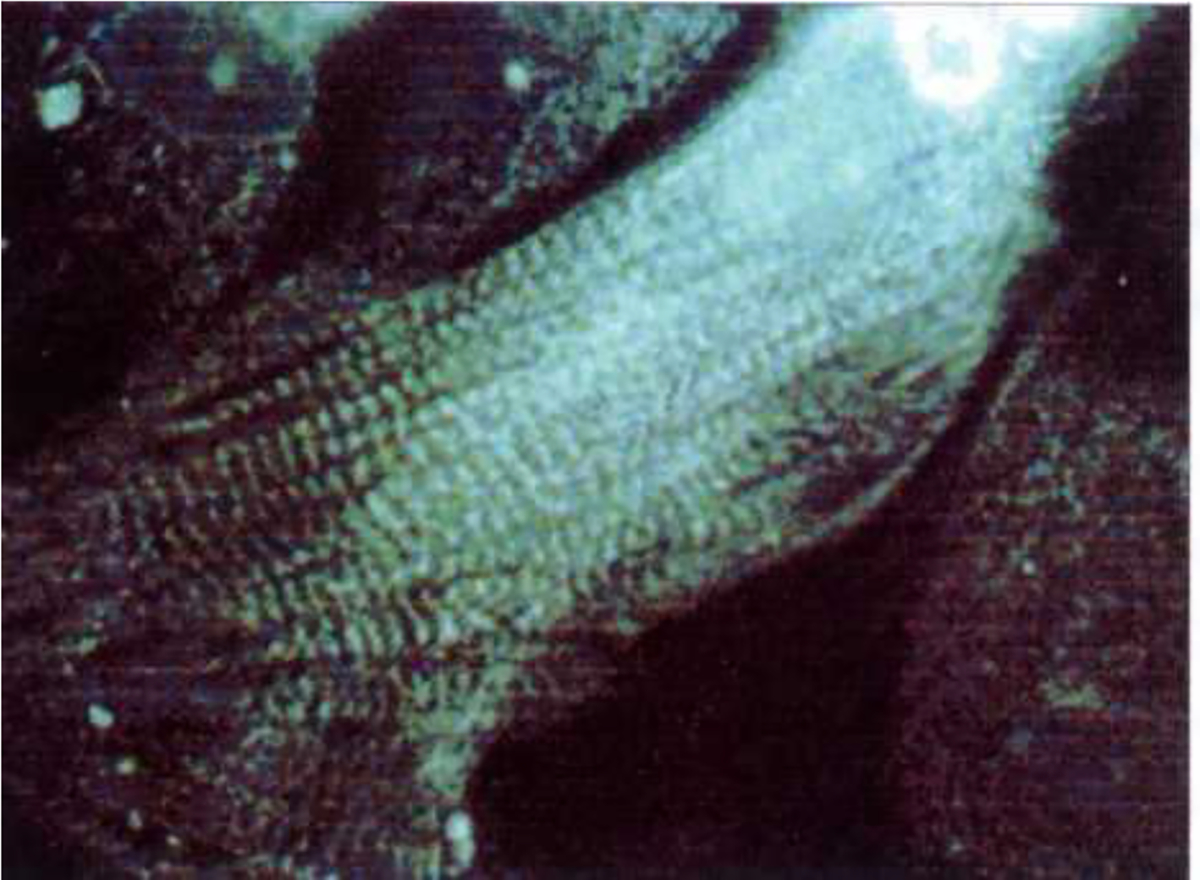
Immunofluorescence staining for α-actinin in a monolayer of cells. Transverse striation of cardiomyocyte contractile structures can be seen (х800).

Enzymatic suspension of fetal myocardial tissue allowed us to obtain 95% of viable cells, the majority of whom were cardiomyocytes (as confirmed by a positive reaction of the cell monolayer with glycogen as a marker of cardiomyocytes in culture, as well as by immunofluorescence staining for alpha-actinin) (Figure 1).

**Figure 2:**
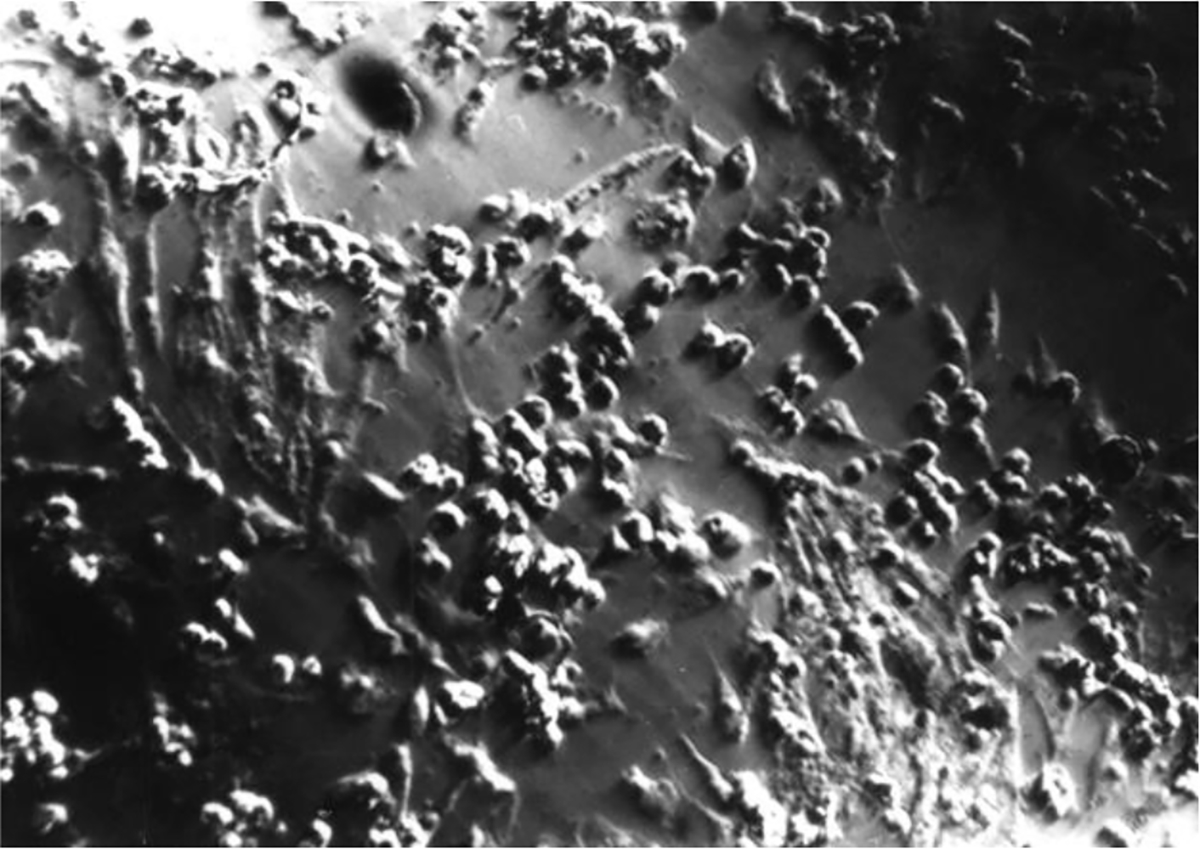
Five-day culture of embryonic cardiomyocytes in an electromagnetic field. Cellular structures are aligned along the momentum propagation vector (the vector is vertical) (x160).

Experiments with cell cultures demonstrated some interesting stages of the gradual arrangements of the syncytium: single attached cells (days 1–2 of incubation); formation of a monolayer and its compaction (days 2–7); formation of multicellular three-dimensional clusters (after day 7). After 3–7 days, we observed electromechanical activity of cells and their conjugates. This activity was initially asynchronous and chaotic; however, after 7 days, we noticed synchronous rhythmic contractions of numerous cell clusters in case of formed communications between them looking like transparent strands (Figure 2).

The main effects of embryonic cell implantation were assessed in a number of experimental models both on cell cultures and animal experiments. Our results convinced us that all of them were ensured by a powerful induction of reparation at the site of lesions. For example, cell cultures exposed to long-term hypoxia demonstrated very high resistance to ischemia after their co-cultivation with a suspension of embryonic cardiomyocytes. These cells appeared unchanged and continued to contract synchronously [6].

**Figure 3:**
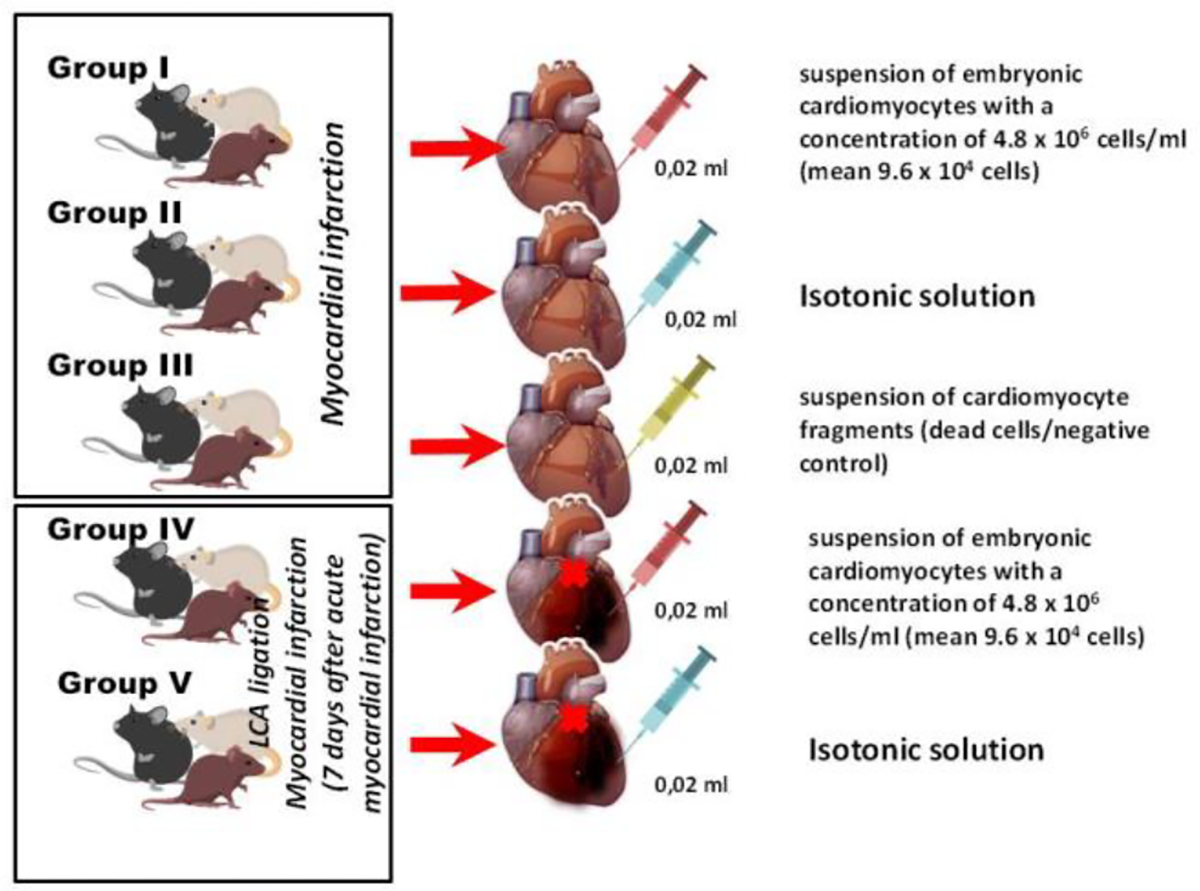
Series of *in vivo* experiments with implantation of embryonic cardiomyocytes in the area of myocardial infarction.

**Figure 4:**
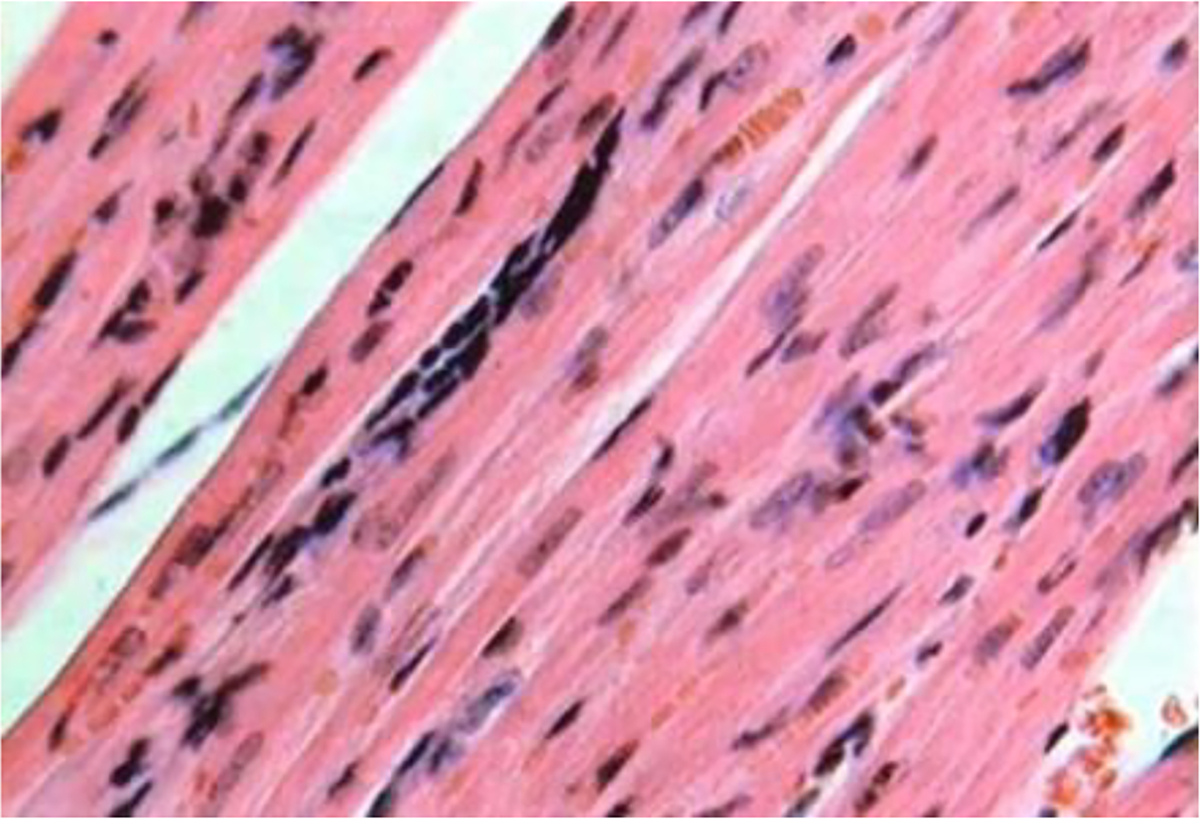
Histological section at the site of embryonic cardiomyocyte implantation in the myocardial infarction area (day 21). Muscle with poorly differentiated cells. Hematoxylin and eosin stain (x200).

We evaluated the condition of embryonic cardiomyocytes implanted in the myocardium in a series of *in vivo* experiments on laboratory rats (Figure 3) and obtained some interesting results. The most important finding was the regression of electrocardiographic signs of acute myocardial injury by day 28 caused by ligation of the left coronary artery branches. Histological examination showed muscle cells at various stages of differentiation (Figure 4).

Positive results of our animal experiments allowed us to use embryonic cardiomyocyte implantation in a patient with severe coronary heart disease. Of note, the use of this technology did not contradict any international and national legal acts existing at that time, which also contributed to such a decision. The case is described below.

## 3. Case Report

**Figure 5:**
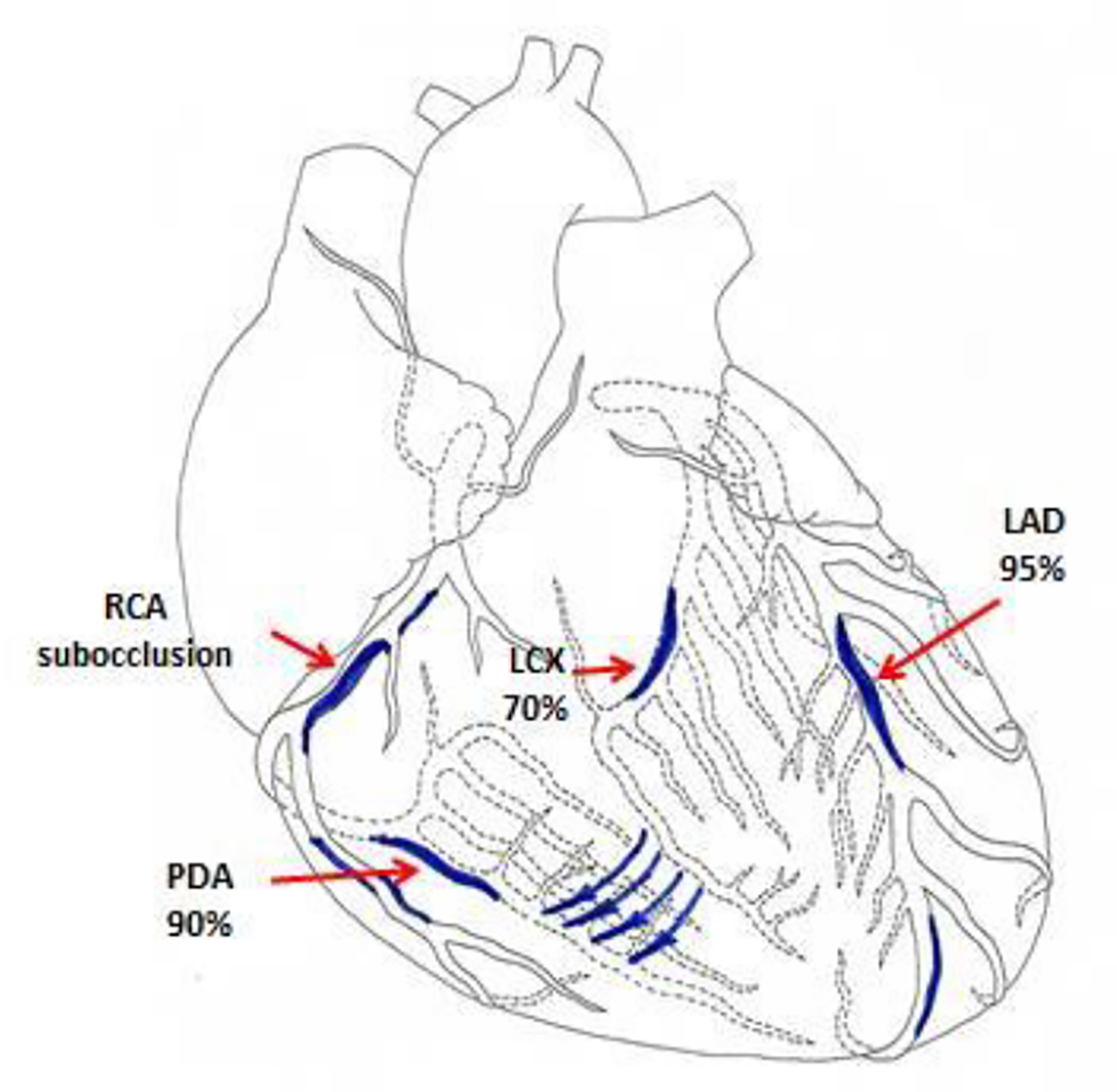
Scheme demonstrating lesions to the coronary bed in a 58-year-old male patient (LAD - Left anterior descending artery, LCX - Left circumflex coronary artery, RCA - Right coronary artery, PDA - Posterior descending artery).

A 58-year-old male patient was admitted to P.A. Kupriyanov Clinic of Cardiovascular Surgery, Military Medical Academy on January 26, 1998 with symptoms of severe angina pectoris and circulatory insufficiency (pain after minor physical exercise, climbing the stairs to the second floor, fast walking for 100–150 m; symptoms were alleviated after taking nitroglycerin). The patient had been sick since 1979 and had two episodes of myocardial infarction: in 1996 and 1997. ECG revealed atrial fibrillation, signs of ischemic lesions, and scarring in the apical part of the septum area and in the anterolateral wall of the left ventricle, which resulted in hypokinesia of the lateral and posterior walls of the left ventricle and decreased total myocardial contractility (ejection fraction 41%). Coronary angiography findings: subocclusion of the left anterior descending artery in its middle third, pathological blood flow to the right coronary artery, occlusion of the right coronary artery in its middle third and 90% stenosis in its distal third, stenosis of the posterior descending artery (Figure 5).

**Figure 6:**
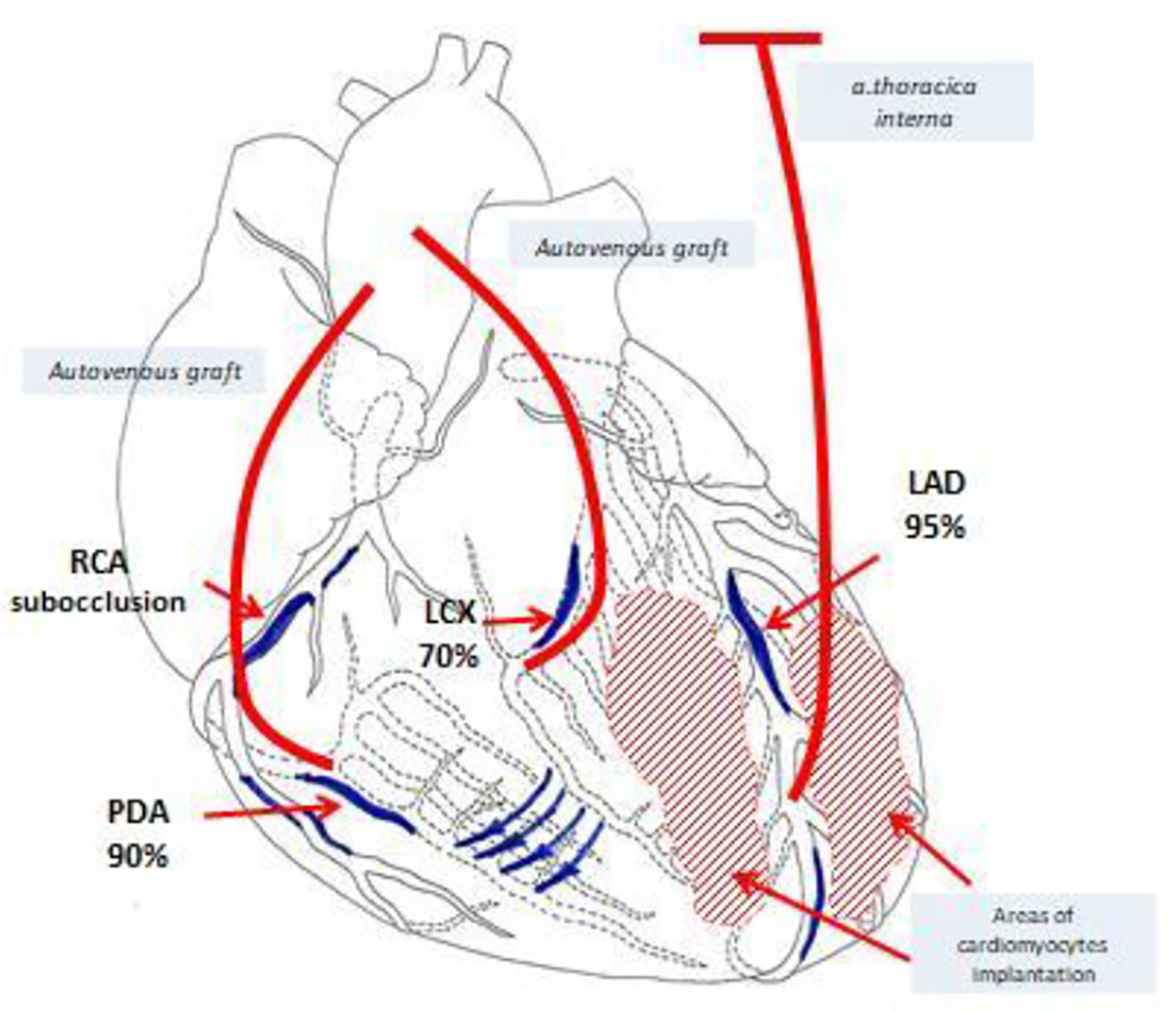
Scheme of surgery (LAD - Left anterior descending artery, LCX - Left circumflex coronary artery, RCA - Right coronary artery, PDA - Posterior descending artery).

Considering extensive damage to the coronary arteries, the type of the disease, and uselessness of conservative therapy, we attempted endovascular recanalization of the left anterior descending artery and right coronary artery on 19.02.1998. However, it was unsuccessful due to the anatomical characteristics of the coronary arteries. We decided to perform direct myocardial revascularization with cardiopulmonary bypass. The severity of coronary lesions with the involvement of peripheral coronary arteries, poor patient’s condition, and ineffectiveness of further conservative therapy led us to decision to use a culture of embryonic cardiomyocytes. We explained the details of the procedure to the patient and he gave his written consent. On 26.02.1998, we performed coronary artery bypass grafting in the affected arteries (three shunts were installed) (Figure 6) with extracorporeal blood circulation and cold cardioplegia.

**Figure 7:**
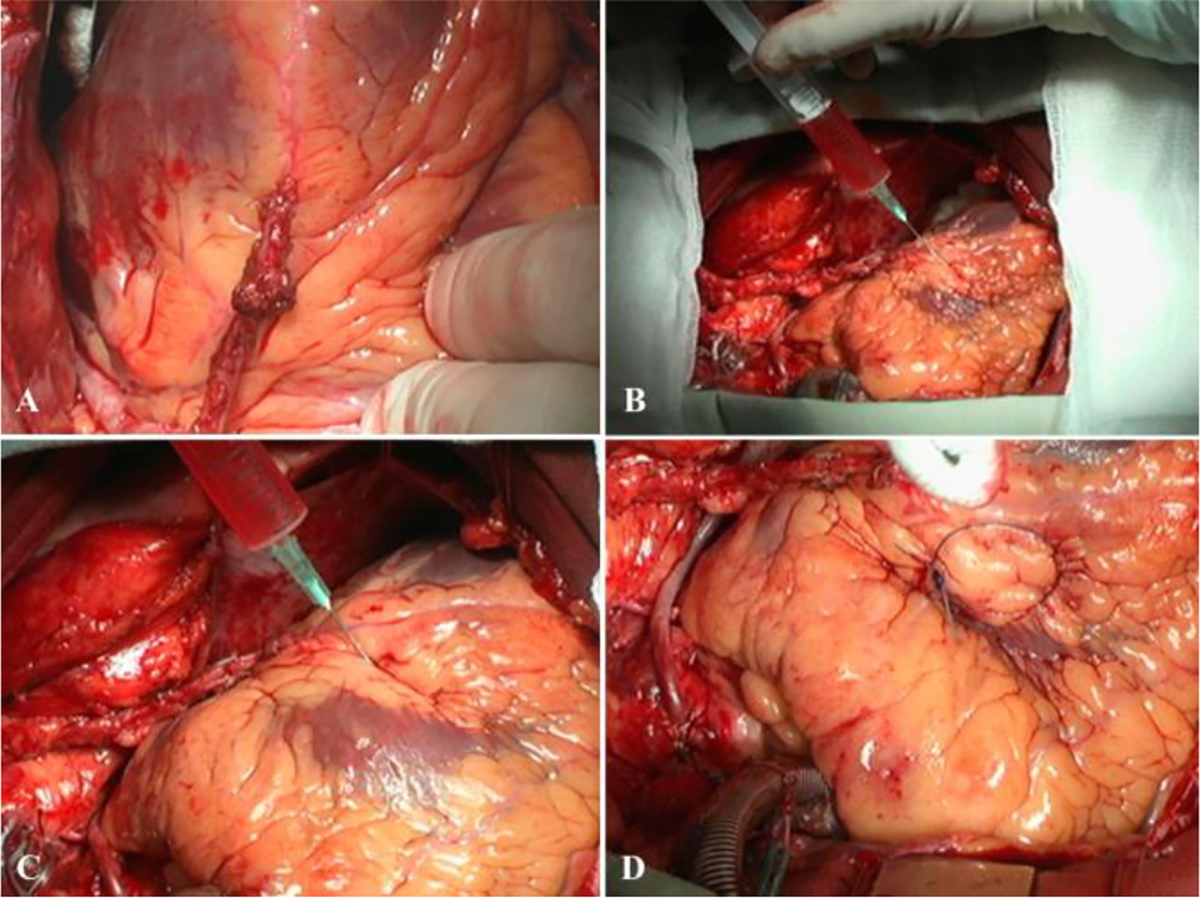
Stages of injection of embryonic cardiomyocyte suspension and marking of one of the implantation areas with a prolene thread. A–myocardial revascularization (mammary coronary and coronary artery bypass grafting (severe sclerotic lesions in the left anterior descending artery–arrows). B, C–stages of injecting a suspension of embryonic cardiomyocytes in the myocardium of the anterolateral wall and the apex. D–marking the implantation area with a prolene thread (mark).

When the main stage of the surgery was completed, hemodynamics stabilized, and cardiopulmonary bypass machine disconnected, we injected a suspension of human embryonic cardiomyocytes into the myocardium of the anterolateral wall and the apex of the left ventricle (4.8 × 10^6^ cells) (Figure 7). The cells were isolated from embryonic material obtained after abortion on 19^th^ week of pregnancy from a woman tested for main infections.

**Figure 8:**
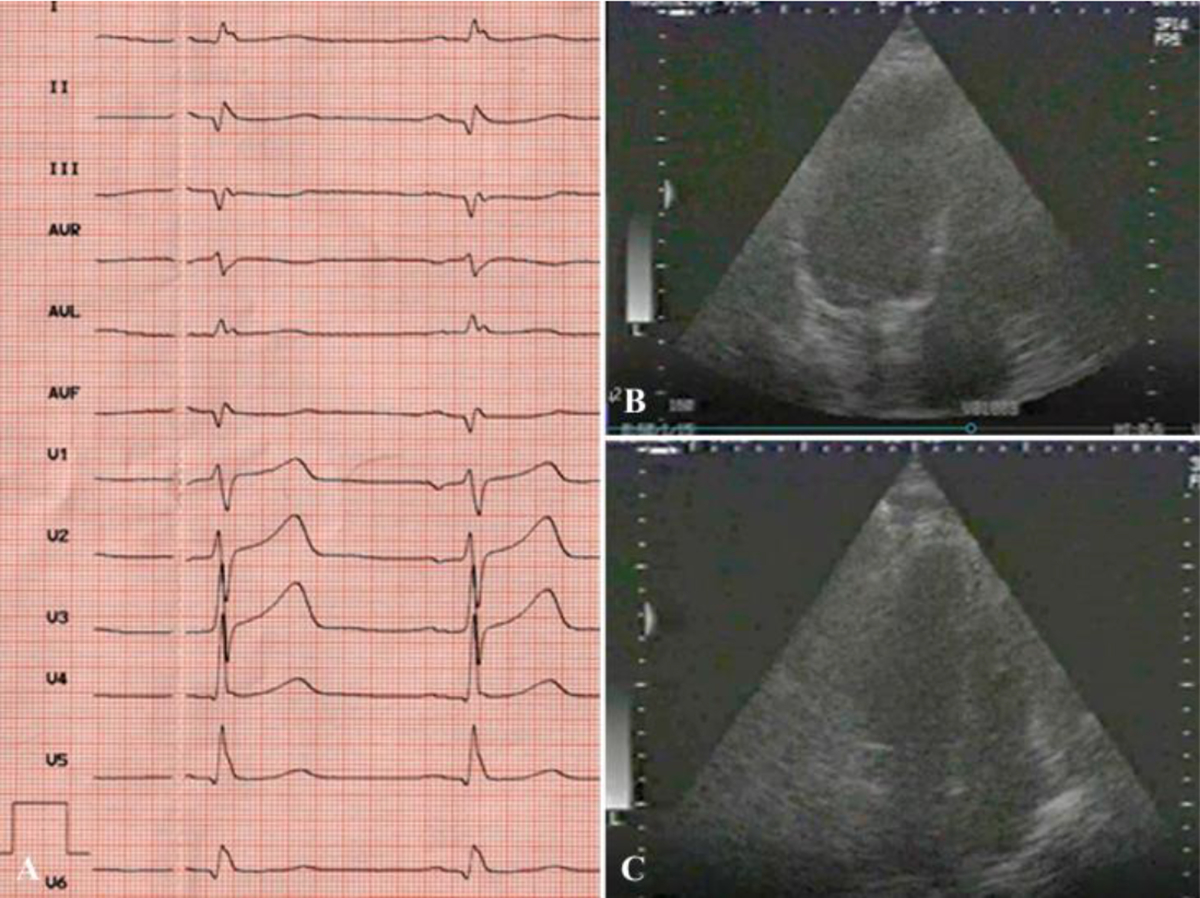
Results of postoperative examination in 2003. ECG (A) and EchoCG (B,C): LV cavity in systole and diastole; good LV myocardial contractility.

The postoperative period was uneventful. The patient was followed-up for a year postoperatively and visited the clinic on a regular basis. He had no symptoms of angina and did not require nitroglycerin. His tolerance to physical exercise increased. Follow-up ECG demonstrated restoration of the sinus rhythm and cicatricial changes in the myocardium. Follow-up echocardiography 5 years postoperatively showed improved contractile function of the myocardium (ejection fraction 56%) (Figure 8). At one of the visits to the clinic in 2003, a video interview was recorded in which he told about it in details. He said that despite being retired, he continues to work at home, in the garden, feels well, tolerates physical exercise, and drives a car.

**Figure 9:**
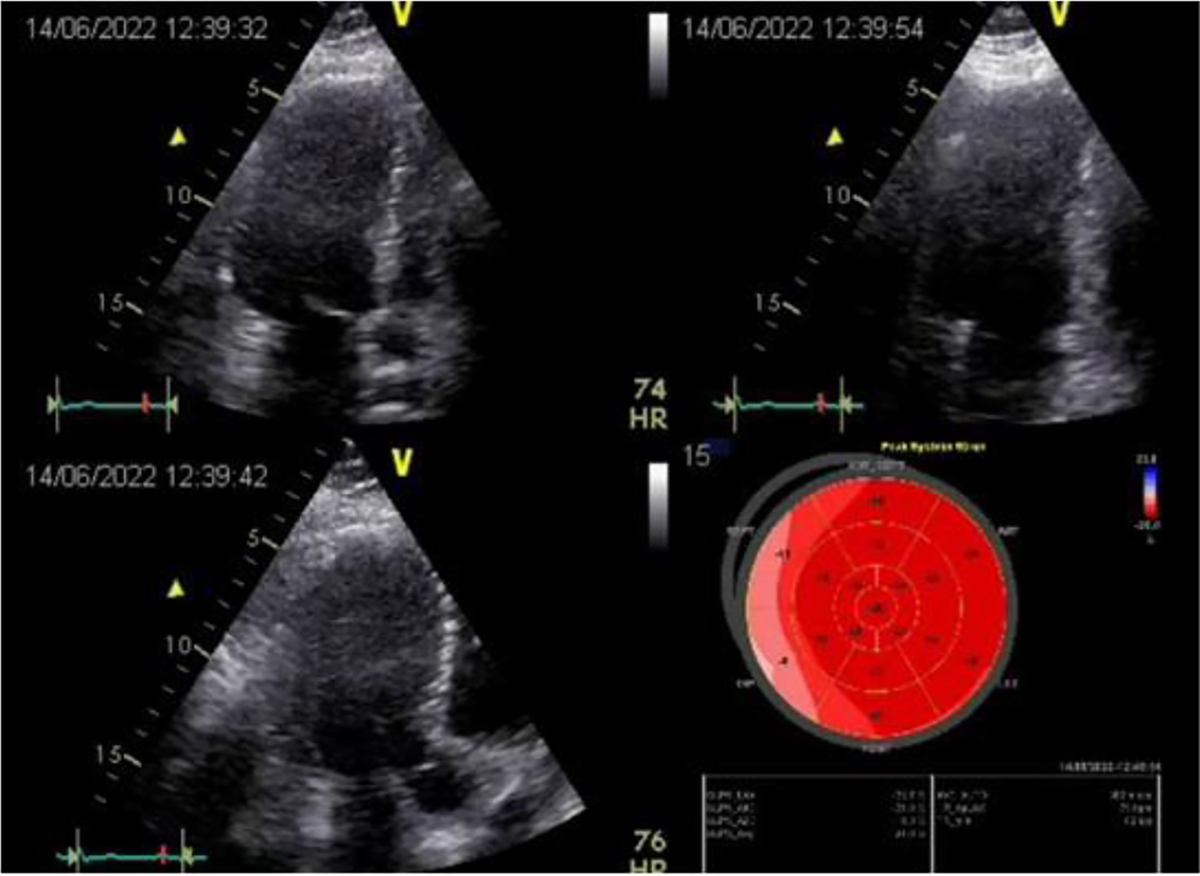
Results of postoperative examination in 2022. EchoCG: good overall contractility of the LV myocardium; EF 54%; signs of minor hypokinesia of the LV lateral wall.

For several years, the patient did not visit the clinic, since he apparently did not have such a need. Twenty-four years following surgery we invited the patient to the clinic to perform comprehensive assessment of the treatment outcome. The information obtained was very useful for assessing the long-term outcome of this operation. Echocardiography (Figure 9) demonstrated satisfactory myocardial contractility (EF 54%) and no local impairments of the myocardial kinetics.

**Figure 10:**
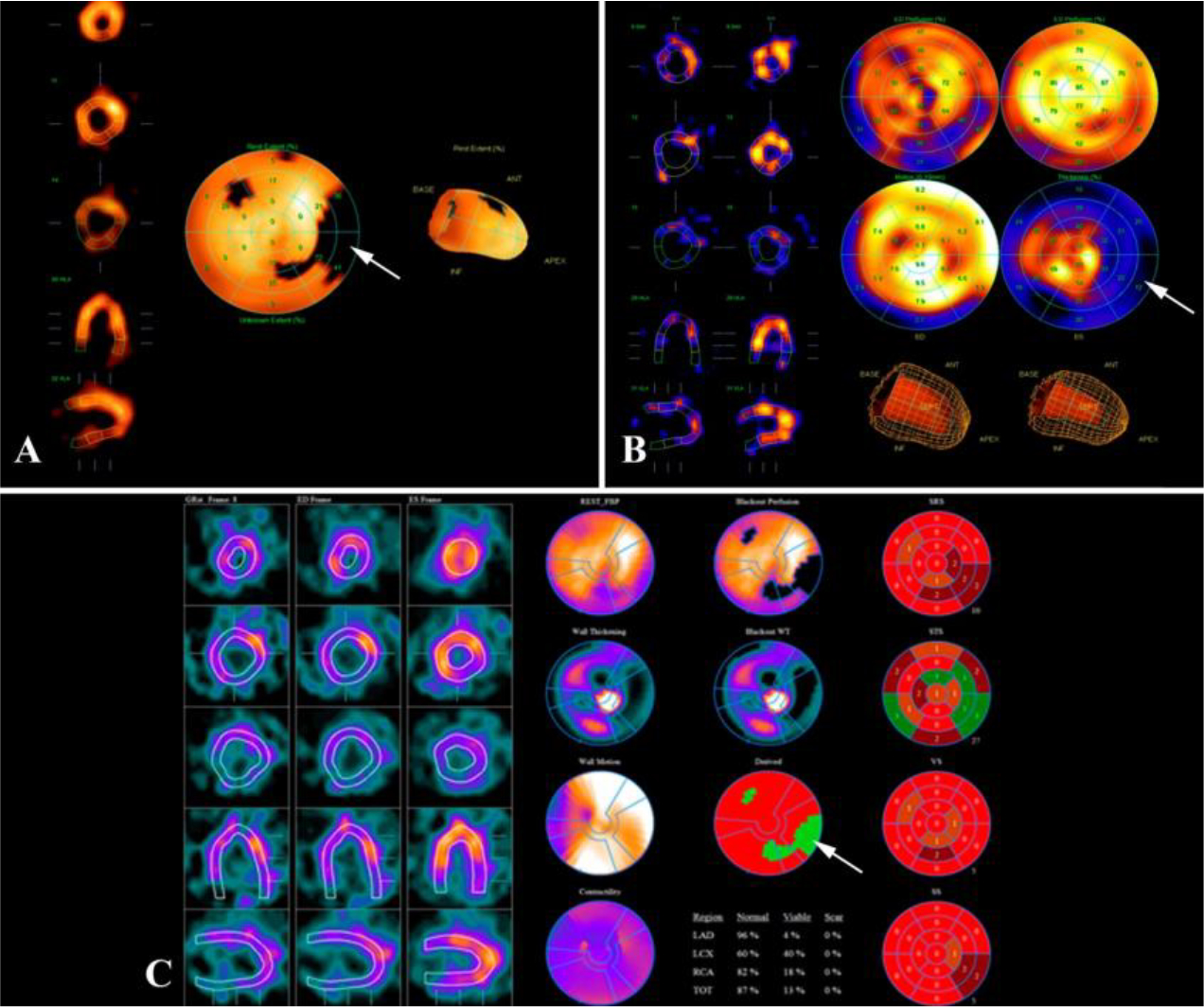
Myocardial scintigraphy at rest. LV myocardial contractility is satisfactory: EF–54%. Perfusion polar diagram of the LV shows a hypoperfusion site in the area of the basal and middle segments of the lateral wall with partial spread to the posterior wall (A, arrow). Hypoperfusion site 13–15%. The hypoperfusion site is characterized by a reduced regional systolic thickening (B, arrow). Differential diagnosis of the type of myocardial lesions in the hypoperfusion site showed the presence of a substantial amount of hibernating myocardium (C, arrow).

The absence of significant ischemic dysfunction was confirmed by scintigraphy. Findings of radionuclide perfusion scintigraphy of the myocardium at rest: LV is slightly enlarged; there are indirect signs of mild myocardial hypertrophy. Contrast agent is unevenly distributed. Moderately reduced contrast agent accumulation in the area of the lateral wall (partially involving basal and middle segments) with the spread to the posterior LV wall at rest. Satisfactory contractility of the LV myocardium; EF 54% (Figure 10).

During this period, the patient worked as a civil engineer for a long time until his retirement in August 2021. He is very active and can easily climb the stairs. Being over 80 years of age, he can walk on the escalator, works at home, at the country house, and feels good. He had COVID-19; currently, he is recovering and taking care of his wife, who had severe COVID-19.

## 4. Discussion

Intramyocardial implantation of cardiomyocytes is a novel and actively developing cell technology. Some authors analyzed the utility of hematopoietic and mesenchymal stem cells to treat different disorders, such as acute myocardial infarction, postinfarction cardiosclerosis, and congestive heart failure. Despite the long history of research and practical attempts, many issues remain to be clarified, in particular, viability of the implanted cells, their integration with the recipient’s myocardium, immunogenicity of cardiomyocytes and its effect on the regenerative potential of their derivatives. Although there are a number of encouraging *ex vivo* studies, experiments with animals have not demonstrated sufficiently good results [7]. It can be due to the differences between cultured cardiomyocytes in terms of nutrient supply, fiber orientation and non-myocytic signaling, differences in the heart physiology between animals and humans, immaturity of transplanted cells, unstructured orientation of myocytes, particularly in the area near the scar [8], etc. The *in vivo* neurohumoral signaling pathways, as well as changes in pre- and post-loading, also affect the heart and, probably, the function of the graft. Another important aspect is an altered immune response in the cardiac tissue after embryonic cardiomyocyte transplantation and development of an inflammatory reaction, the characteristics of which are important for predicting both functional outcome of the procedure [8] and the risk of immobilizing cardiac fibrosis [9]. Immunomodulation is one of the main effects of mesenchymal stem cell transplantation [10]. Embryonic cardiomyocyte transplantation reduces the number of macrophages and T cells during acute myocardial infarction [8], encompassing both acute and chronic phases of myocardial infarction. Researchers from the University of Toronto [11] demonstrated that macrophages are involved in both damage and recovery after myocardial infarction; however, distinguishing their functions in mixed populations remains challenging. Ischemic damage reduced the number of tissue-resident macrophages TIMD4+ and TIMD4, whereas macrophages derived from CCR2+ monocytes differentiated in necrotic tissues into the cells almost indistinguishable from tissue-resident macrophages. Depletion of tissue-resident macrophages led to an impaired cardiac function and triggered unfavorable remodeling, primarily in the periinfarction zone, corroborating the cardioprotective role of resident cardiac macrophages. Thus, prevention of monocyte migration to the cardiac tissue may impede their subsequent development into macrophages with reparative functions. Studies on embryonic cardiomyocyte differentiation with tissue matrices [12,13] also demonstrated the paracrine effects of implanted cells in addition to their ability to contract, as well as the existence of certain circadian metabolic processes probably underlying the development of cellular automatism [14]. This data can be helpful for choosing an optimal cell type for cell therapy. The absence of capillary enlargement and a decrease in the area of infarction, observed by Vasudevan et al. (2020), suggests that cardiomyocyte transplantation alone might be insufficient for proper recovery and regeneration of the affected heart; the effect of cell implantation on these processes requires further investigation [8]. To our knowledge, the case described in this article is one of the first reports of embryonic cardiomyocyte implantation into the human heart. Since the time of this surgery, our team has reconsidered the position on this problem along with accumulation of experimental and clinical information. We have no illusion that implanted cardiomyocytes are integrated into the structure of the myocardium and are included in the overall function of the heart. Most probably, the laws of biology are not in favor of this hypothesis. Presumably, aggressive local factors caused death of the implanted cells. However, the derivatives and cellular components containing growth factors acted as powerful stimulators of both angiogenesis and, possibly, activators of mitosis in patient’s cardiomyocytes, promoting revascularization of ischemic heart tissue. Evidently, extracardial myocardial revascularization resulted from sternotomy, pericardiotomy, and natural aseptic inflammation of mediastinal tissues and stimulated by growth factors and tissue derivatives of embryonic cardiomyocytes, also had some role. Long-term follow-up and assessment of the treatment outcome of our patient was important in creating the concept of extracardial revascularization, described in the recently published article «Extracardial myocardial revascularization in patients with coronary heart disease and diffuse coronary lesions» [9].

## 5. Conclusion

We believe that cell therapy is possible and its prospects are undoubted, whereas its cancer risk is probably exaggerated. The case described here is the only one in our study. We have not tried to perform implantation of human embryonic cardiomyocytes for a second time due to the ethical and legal problems, but continued similar studies on animal models.
